# Trajectories of adolescent perceived stress and symptoms of depression and anxiety during the COVID-19 pandemic

**DOI:** 10.1038/s41598-022-20344-y

**Published:** 2022-09-24

**Authors:** Amanda W. G. van Loon, Hanneke E. Creemers, Simone Vogelaar, Nadira Saab, Anne C. Miers, P. Michiel Westenberg, Jessica J. Asscher

**Affiliations:** 1 Child and Adolescent Studies, Utrecht University, Heidelberglaan 1, 3584 CS Utrecht, The Netherlands; 2 Forensic Child and Youth Care Sciences, University of Amsterdam, Nieuwe Achtergracht 127, 1018 WS Amsterdam, The Netherlands; 3 Developmental and Educational Psychology, Leiden University, Wassenaarseweg 52, 2333 AK Leiden, The Netherlands; 4 Graduate School of Teaching (ICLON), Leiden University, Kolffpad 1, 2333 BN Leiden, The Netherlands

**Keywords:** Human behaviour, Public health, Paediatric research

## Abstract

Adolescents might be particularly affected by the drastic social changes as a consequence of the COVID-19 pandemic, given the increased stress-sensitivity and importance of the social environment in this developmental phase. In order to examine heterogeneity during the pandemic, the current study aimed to identify whether subgroups of adolescents could be distinguished based on their levels of perceived stress and symptoms of depression and anxiety. In addition, we examined which prepandemic factors predicted these trajectories. Adolescents were assessed before the pandemic (*N* = 188, *M*_age_ = 13.49, *SD* = 0.81) and at three timepoints during the pandemic (i.e., eight, ten, and 15 months after the start of the pandemic in the Netherlands). Results showed no support for distinct trajectories of perceived stress, adolescents experienced stable moderate levels during the pandemic. In contrast, results showed three trajectories for depression and anxiety. The majority of adolescents reported stable low or moderate levels and one small subgroup reported high levels of depression and anxiety that decreased during the pandemic. Certain prepandemic factors predicted higher initial levels of stress and symptoms of depression and anxiety during the pandemic. To support adolescents with prepandemic vulnerabilities, strategies could be developed, for instance enhancing adolescents’ social support.

## Introduction

The coronavirus disease 2019 (COVID-19) pandemic and national measures to prevent the spread of the virus have profoundly disrupted the daily (social) lives of individuals. In the Netherlands for instance, from March to May 2020, the government enforced restrictions such as social distancing and closing of public spaces, including closing of schools (i.e., first lockdown). Between December 2020 and February 2021, public spaces were closed again, as well as non-essential shops (i.e., second lockdown). Further, in the time between the lockdowns and after, ongoing measures were in effect to limit social interactions (e.g., working from home, limiting group sizes). Adolescence is a period of elevated stress-sensitivity and a period where the social environment is of increasing importance, especially peer interaction^1–3^. As such, adolescents might be particularly affected by the drastic (social) changes as a consequence of the COVID-19 pandemic, in particular the fewer opportunities for physical peer interaction, the changing demands regarding school, and the uncertainty about the (course of) the pandemic. In previous studies adolescents indeed reported concerns about the COVID-19 pandemic, specifically related to social activities and school^4–6^. In addition, recently published longitudinal studies conducted at the beginning of the COVID-19 pandemic observed increased distress, depressive, and anxiety symptoms in adolescents from before to during the pandemic^5,7–9^, suggesting a negative impact of the pandemic on the mental health of adolescents.

Although previous longitudinal studies provide insight into the development of mental health problems during the first months of the COVID-19 pandemic, they focus on the average pathway for adolescents (i.e., group means), and do not allow for identifying subgroups of individuals based on mental health changes over time. Yet, it is likely that not all adolescents experience COVID-19-related mental health changes to the same extent, or in the same direction^10^. For instance, some adolescents might benefit from the enforced measures. In fact, a recent study observed that almost a quarter of adolescents became more satisfied with their life at the beginning of the COVID-19 pandemic^11^, likely because of perceived benefits from staying at home (e.g., more time for family and personal activities). Furthermore, studies investigating moderators of changes in mental health from before to during the pandemic, such as gender and living condition, demonstrated different subgroups of adolescents in terms of how they respond to the COVID-19 pandemic^5,12^, also pointing towards differential trajectories of mental health changes over time.

In addition to identifying differential trajectories, identifying predictors of adverse trajectories (e.g., characterized by an increase or high level of problems) can help recognize adolescents in need of help and support. For instance, adolescents with higher levels of perceived stress and symptoms of depression and anxiety before the pandemic might be more susceptible to negative consequences of the pandemic, as these adolescents tend to worry about uncertain and unpredictable situations. Indeed, previous research in adolescents demonstrated that having mental health problems before the pandemic predicted higher levels of anxiety and depressive symptoms during the pandemic^13^. Moreover, previous findings of the current study’s sample indicated that adolescents with specific prepandemic vulnerabilities (i.e., higher stress, maladaptive coping, and internalizing problems) experienced more COVID-19-related concerns during the pandemic^6^. Besides prepandemic levels of perceived stress and symptoms of depression and anxiety, demographic characteristics may be relevant predictors of mental health trajectories during the COVID-19 pandemic. Findings from previous research, mostly focusing on changes from before to during the pandemic, suggest that female gender^5,13^, educational level^14^, ethnic minority background^12,14^, and living in a single parent household^13^ are associated with stronger increases in internalizing problems. For age, previous findings are mixed^14–16^. Finally, psychosocial factors, including self-esteem and social support, seem important to consider as potential predictors of change in adolescent mental health. Previous research suggests that self-esteem and social support protect against experiencing negative effects from the COVID-19 pandemic^9,17,18^.

Studies with longitudinal designs and prepandemic measures are necessary to determine long-term effects on indicators of mental health and to comprehensively apprehend the multiple factors playing a role^19–21^. Different trajectories of change in mental health during the COVID-19 pandemic have not yet been examined in adolescents. The current study, therefore, aimed to investigate trajectories of mental health change in adolescents during the COVID-19 pandemic, by focusing on perceived stress and symptoms of depression and anxiety as indicators of mental health. Adolescents were followed up to more than a year into the pandemic, with assessments at 8, 10, and 15 months after the start of the pandemic in the Netherlands. The first aim was to explore which trajectories could be distinguished in the course of perceived stress and symptoms of depression and anxiety during the pandemic. Given that moderator studies in adolescents observed different subgroups in response to the COVID-19 pandemic^5,12^, we expected heterogeneity in changes in perceived stress and symptoms of depression and anxiety among adolescents during the pandemic. The second aim was to investigate which prepandemic factors (i.e., stress, depression and anxiety, demographics, and psychosocial variables) predicted these trajectories. We expected that adolescents with higher prepandemic levels of perceived stress and symptoms of depression and anxiety, female adolescents, adolescents with a lower educational level, adolescents with a migrant ethnic identity, adolescents that do not live with both of their parents, and adolescents with lower self-esteem and social support before the pandemic are more likely to experience adverse courses of perceived stress and symptoms of depression and anxiety during the COVID-19 pandemic. Examining heterogeneity of changes in mental health indicators during the COVID-19 pandemic can add to our understanding of how adolescents experience and deal with the pandemic. Insight in these changes can provide knowledge about the implications of the national measures (e.g., social distancing, closing of public spaces), which might prepare governments for future developments regarding the pandemic or possible other crises. Moreover, by identifying subgroups of adolescents that show differential trajectories of change in perceived stress and symptoms of depression and anxiety during the pandemic and identifying factors that predict these trajectories, appropriate support could be arranged for adolescents with adverse courses, by providing individualized and tailored care.

## Methods

### Study design, participants, and procedure

Data were collected within the context of an ongoing school-based intervention effectiveness study (i.e., randomized controlled study) conducted in secondary schools located in one of the four largest cities in the Netherlands. The COVID-19 outbreak occurred during the course of this study, which provided an ideal opportunity to investigate the impact of the COVID-19 pandemic on indicators of mental health in adolescents. The effectiveness study examined two school-based skills-training programs in which adolescents enrolled by self-selection, addressing social skills or skills to deal with performance anxiety^22^. The small-group skills-training programs consisted of seven sessions of 45 min. As secondary schools were closed due to the outbreak of COVID-19, the skills-training programs that started in February 2020 were cancelled after some initial sessions (i.e., 2–4 sessions). The programs restarted in the next school year (i.e., at four schools, with the same students). Students enrolled in the effectiveness study in February 2020 (*N* = 188) constitute the sample of this study.

**Figure 1:**
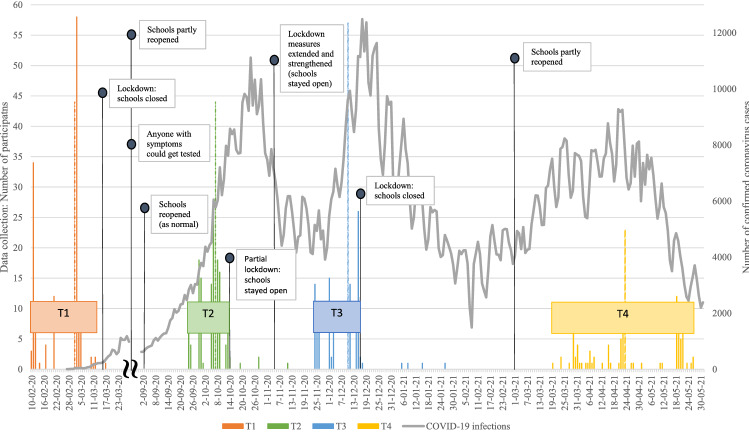
Overview of the data collection windows, the number of confirmed coronavirus cases and the government restrictions in the Netherlands. Note. The histograms present the number of assessed participants per timepoint, for each data collection window (T1: orange, T2: green, T3: blue, and T4: yellow). The dashed lines show the median of the data collection window. The line represents the number of confirmed coronavirus infections per day (adapted from the National Institute for Public Health and the Environment [RIVM])^49^. From June 1, 2020, everyone with coronavirus-symptoms could get tested. Specific government restrictions regarding secondary schools are represented in text (adapted from the RIVM)^43^.

Figure 1 presents an overview of the windows of data assessment, as well as infection rates and government measures in the Netherlands. Pre-COVID-19 pandemic data were collected between 10 February and 17 March 2020, before the first COVID-19 lockdown in the Netherlands (T1). During the COVID-19 pandemic, data were collected 8 months after the first measurement (T2; on average 31 weeks after T1), 2 months after the second measurement (T3; on average 9 weeks after T2), and 5 months after the third measurement (T4; on average 19 weeks after T3). In total, the assessments covered a period from before the COVID-19 pandemic, to 15 months into the pandemic (i.e., over a year later). The first measurement (T1) occurred before the COVID-19 pandemic restrictions were put into effect, and students were at school. The second measurement (T2) took place during a period of fewer restrictions, when students were at school again (i.e., after the first lockdown when schools were closed and after the summer holiday). The third measurement (T3) was during a period of stricter restrictions (i.e., after the situation deteriorated again), but students were still at school. The fourth measurement (T4) took place during a period of fewer restrictions, but schools were only partly open and most students were receiving online education (Fig. 1).

The sample consists of 188 adolescents (102 females; 54.3%) aged between 12.2 and 15.9 years, with a mean age of 13.49 years (*SD* = 0.81). Most participants were first year secondary school students (65.4%, equivalent to USA 7th grade) before the pandemic (T1), and were in their second year during the pandemic (at T2, T3, and T4). The rest of the participants were second or third year secondary school students (34.6%, equivalent to USA 8th and 9th grade) at T1. The sample consisted of prevocational students (i.e., lowest educational level; 32.4%), prevocational/senior general education students (26.6%), and senior general education-preuniversity education students (i.e., highest educational level; 41.0%). Most of the participants reported the Netherlands as country of birth (93.1%). Contrary to country of birth, the ethnic identity of participants was more mixed (Western, 60.6%, for instance: Dutch or Polish; Mix Western-non Western, 17.6%, for instance: Dutch-Turkish or Dutch-Surinamese; and Non Western, 21.8%, for instance: Moroccan or Turkish). More than half of the participants (66.0%) reported that they lived with both their parents, while the other participants (34.0%) reported something else (e.g., living with only one parent, not living with either parent). Adolescents had an average of 1.76 siblings (*SD* = 1.28, ranging from 0 to 9 siblings). Ten participants reported that their family had financial problems (5.3%). The number of participants per data collection wave was 188 (T1), 178 (T2), 170 (T3), and 117 (T4). The retention rate from T1 to the other waves was respectively 94.7% (T1–T2), 90.4% (T1–T3), and 62.2% (T1–T4).

Independent t-and chi-square tests were performed to investigate differences at T1 between participants and dropouts at T2, T3, and T4. There were no statistically significant differences in mental health indicators (i.e., stress, depression and anxiety), demographics (e.g., age, gender, educational level, ethnic identity, and living situation), and psychosocial variables (i.e., self-esteem and social support) at T1 between the current sample and dropouts at T2 (*N* = 10), at T3 (*N* = 18) or at T4 (*N* = 71), except for educational level and country of birth. Dropouts at T4 were more likely to have a lower educational level (χ^2^(2) = 41.147, *p* < 0.001) and were more often born in another country than the Netherlands (χ^2^(1) = 5.883, *p* = 0.020). One school did not want to participate in the fourth measurement wave (T4), which might explain the differences in educational level, as this school educated adolescents at the lowest educational level only (*N* = 21).

As students were enrolled in school-based skills-training programs immediately after T1 (i.e., experimental group), between T2 and T3 (i.e., restarted programs; experimental group) and between T3 and T4 (i.e., control group), correlation analyses were performed to examine potential associations between number of attended sessions and mental health indicators (i.e., stress, depression and anxiety). Number of attended training sessions between T1 and T2 and between T2 and T3 were not associated with perceived stress and symptoms of depression and anxiety (at T2 and T3, respectively). Number of attended training sessions between T3 and T4 was positively associated with perceived stress and symptoms of depression and anxiety at T4 (*r* = 0.342 and *r* = 0.307, respectively). After combining the groups, number of attended training sessions between T1-T4 (*N* = 112 participants attended more than one session) was also positively related to perceived stress and symptoms of depression and anxiety at T4 (*r* = 0.410 and 0.374, respectively). To control for this positive correlation, we added number of attended training sessions (between T1-T4) as predictor in the analyses. Almost half of the participants (*N* = 76) did not attend any training session.

Assessments at T1 and T2 were at school. At T3, most assessments were again at school, but a small number of students were assessed online, as schools had to close suddenly (*N* = 7, 4%). At T4, assessments were both online and at school. To motivate students to fill in the questionnaire again, students received a compensation of six euros after completion of the assessment (i.e., at T4 only). Unfortunately, because of the online assessment and partial lockdown, it was difficult to reach all students. Hence, the attrition rate was relatively high (37.8%) for the fourth measurement wave. Active parental and student informed consent was obtained for the students’ participation in the study. The Psychology Ethical Committee of Leiden University approved the study (CEP19-1210/577). The study was performed in accordance with relevant guidelines and regulations (Declaration of Helsinki) for research involving human participants.

### Instruments

#### Mental health indicators

##### Perceived stress

The Chronic Stress Questionnaire for Children and Adolescents (CSQ-CA) was used to assess perceived stress levels^23^. Adolescents completed the questionnaire consisting of 17 items (e.g., “I often get upset about things that are not important”) rated on a 4-point Likert scale from 1 (*not true for me at all*) to 4 (*completely true for me*). A higher score reflects more stress (α = 0.79 at T1, α = 0.85 at T2, α = 0.85 at T3, and α = 0.84 at T4).

##### Depression and anxiety

The Dutch version of the Youth Outcome Questionnaire (Y-OQ-30.1) was used to measure depression and anxiety symptoms^24^. The subscale Depression/Anxiety, consisting of 6 items, was used (e.g., “I am sad or unhappy” or “I am worried that I cannot get thoughts out of my head”). Adolescents reported their depression and anxiety symptoms on a 5-point Likert scale from 0 (*never*) to 4 (*always*). A higher score reflects more symptoms of depression and anxiety (α = 0.76 at T1, α = 0.85 at T2, α = 0.87 at T3, and α = 0.82 at T4).

#### Potential predictors of change

##### Demographics

Adolescent characteristics were collected at baseline (T1), which included age, gender, educational level, ethnic identity, and living situation. *Ethnic identity* was assessed by asking the participants with which identity they felt most connected to (I see myself as: “Dutch, Indonesian, Turk, Moroccan, Surinamese, Antillean, or other”). Three groups were distinguished: Western (e.g., Dutch), mix Western-non Western (e.g., Dutch-Moroccan) or Non Western (e.g., Turkish). *Living situation* was assessed by asking the participants about their living situation (Indicate what is right for you, with answer options such as “I live with both my parents, and my parents live together in one house”, “I live alone with my father”, “I don’t live with any of my parents”, or “other”). Two groups were distinguished: Adolescents who lived with both their parents in the same house and adolescents that reported something else.

##### Self-esteem

The Dutch version of the Rosenberg Self-Esteem Scale (RSES) was used to assess self-esteem^25,26^. The instrument consists of 10 items (e.g., “At times I think I am no good at all”) rated on a 4-point Likert scale from 1 (*strongly agree*) to 4 (*strongly disagree*). A higher score reflects more self-esteem (α = 0.86 at T1).

##### Social support

The Social Support List—Interactions (SSL12-I) was used to measure the extent of social support received by social interactions in adolescents’ network^27^. Adolescents reported how often a situation happened to them (e.g., Does it ever happen to you that people: “Are interested in you?” or “Ask you for help or advice”?) rated on a 4-point scale from 1 (*seldom or never*) to 4 (*very often*). The scale consists of 12 items, with a higher score reflecting more social support (α = 0.92 at T1).

#### Statistical analyses

Descriptive and correlation analyses for included variables were performed using SPSS version 25. To examine trajectories concerning change in adolescent mental health indicators (i.e., stress, depression and anxiety) during the COVID-19 pandemic, a Growth Mixture Model (GMM) was conducted using M*plus* version 8.7. GMM is useful for identifying homogenous subgroups within the larger heterogenous population, by estimating growth curves for each class and allowing individual variation around these growth curves^28^. Perceived stress and symptoms of depression and anxiety were analyzed separately.

First, Latent Growth Curve (LGC) modeling was used to examine the linear change in perceived stress and symptoms of depression and anxiety from T2 to T4 (i.e., during the pandemic) for the whole group. The intervals between timepoints were adjusted to fit our timeline (i.e., 2 months between T2 and T3 and 5 months between T3 and T4). A Comparative Fit Index (CFI) and Tucker-Lewis Index (TLI) above 0.95, a Root Mean Square Error of Approximation (RMSEA) below 0.10, and a Standardized Root Mean Square Residual (SRMR) below 0.08 reflect a good model fit^29^. Second, Latent Class Growth Analyses (LCGA)[The LCGA results are available on request] were performed as precursors of the GMM analyses, given that LCGA models are less complex as only variance between classes (and not within classes) is allowed^28^. Third, GMM analyses were conducted with an increasing number of classes, up until the new model (with an extra class) did not outperform the previous model, based on the Bayesian Information Criteria (BIC). Missing data were handled using a full-information maximum likelihood estimator with standard errors (MLR), that is robust to non-normality in the variables^28^. Random starts and start iterations were increased to ensure successful convergence. The best-fitting model was chosen based on a low BIC, a high entropy (> 0.80)^30^, a significant improvement in model fit based on the Vuong-Lo-Mendell-Rubin Likelihood Ratio Test (VLMR-LRT) and Bootstrapped Likelihood Ratio test (BLRT), the size of the subgroups, theoretic justification, and interpretability of the observed trajectories^28^.

To examine predictors of the trajectories, the prepandemic factors (i.e., stress, depression and anxiety, age, gender, educational level, ethnic identity, living situation, self-esteem and social support) were added to the model. A three-step method was used to predict class membership^31^, which takes into account the uncertainty or misclassification of class membership. If the entropy is high enough, the application of the three-step method is fairly reliable^31^. As described above, the model was estimated (step 1) and the most likely class membership (i.e., the probability of belonging to one of the trajectories) was determined (step 2) without adding the predictors. The predictors were simultaneously added to the model (step 3), and using logistic regression, class membership was predicted.

## Results

Table 1 presents the correlations and descriptive statistics at T1, T2, T3, and T4 for all study variables.

**Table 1:** Correlations and descriptive statistics (means, standard deviations, ranges, and sample sizes) for study variables.

	1	2	3	4	5	6	7	Mean	*SD*	Min–max	*N*
1. Age (T1)		–						13.49	0.81	12–16	188
2. Gender (T1)^a^	− .02	–						–	–	1–2	188
3. Educational level (T1)^b^	.19**	− .08	–					–	–	1–3	188
4. Ethnic identity (T1)^c^	− .09	.05	− .36***	–				–	–	1–3	188
5. Living situation (T1)^d^	− .05	.05	− .13	− .07	–			–	–	1–2	188
6. Self-esteem (T1)	.03	− .17*	.02	.02	− .11	–		2.85	0.59	1–4	188
7. Social support (T1)	− .01	.14	− .01	− .06	.07	.44***	–	2.86	0.67	1–4	187
T1								T1			
8. Stress (T1)	.07	.13	.04	.02	.20**	− .37***	− .20**	2.42	0.43	1–4	188
9. Depression/anxiety (T1)	− .01	.14	− .07	.00	.18*	− .68***	− .40***	1.12	0.77	0–4	187
T2								T2			
8. Stress (T2)	.13	.18*	.04	− .08	.23**	− .33***	− .28***	2.37	0.49	1–4	178
9. Depression/anxiety (T2)	.05	.15	.00	− .09	.18*	− .52***	− .41***	1.10	0.88	0–4	178
T3								T3			
8. Stress (T3)	.06	.22**	− .01	− .01	.17*	− .29***	− .22**	2.39	0.50	1–4	170
9. Depression/anxiety (T3)	.04	.21**	− .06	− .08	.19*	− .49***	− .34***	1.13	0.91	0–4	170
T4								T4			
8. Stress (T4)	.10	.11	.12	− .01	.27**	− .22*	− .18	2.34	0.47	1–3	116
9. Depression/anxiety (T4)	.04	.12	.03	− .01	.23*	− .41***	− .29**	1.06	0.79	0–3	116

Significant correlations are bolded. For gender, educational level, ethnic identity, and living situations, Spearman correlations were conducted.

***p < .001; **p < .01; *p < .05.

^a^1 = male, 2 = female.

^b^1 = prevocational educational level, 2 = prevocational / senior general educational level, 3 = senior general / preuniversity educational level.

^c^1 = Western, 2 = mixed Western-non-Western, 3 = non-Western.

^d^1 = living situation with both parents, 2 = other.

### Change in perceived stress and symptoms of depression and anxiety

LGC analyses were performed to examine whether perceived stress and symptoms of depression and anxiety changed during the COVID-19 pandemic (Table 2). The linear growth model for stress demonstrated a good fit, CFI = 0.996, TLI = 0.989, RMSEA = 0.070, SRMR = 0.018. The linear growth model for depression and anxiety also demonstrated a good fit, CFI = 1.000, TLI = 1.000, RMSEA = 0.000, SRMR = 0.012. Overall, both perceived stress and symptoms of depression and anxiety demonstrated a stable course during the pandemic, that is, no significant slopes were observed (Table 2).

**Table 2:** The model fit information and descriptives for the LGC models for stress and depression and anxiety (N = 178).

	AIC	Adjusted BIC	BIC	RMSEA	CFI	TLI	SRMR	Intercept *M* (*SE*), *p*	Slope *M* (*SE*), *p*	Intercept V (*SE*), *p*	Slope V (*SE*), *p*
Stress	432.363	432.483	457.818	0.070	.996	.989	0.018	2.378 (0.037), < .001	− 0.001 (0.013), .941	0.193 (0.029), < .001	0.008 (0.008), .368
Depression/anxiety	979.409	979.529	1004.864	0.000	1.000	1.000	0.012	1.110 (0.065), < .001	0.002 (0.022), .917	0.618 (0.097), < .001	0.017 (0.029), .549

*LGC* Latent Growth Curve, *AIC* Akaike Information Criterion, *BIC* Bayesian Information Criterion, *RMSEA* Root Mean Square Error of Approximation, *CFI* Comparative Fit Index, *TLI* Tucker–Lewis Index, *SRMR* Standardized Root Mean Square Residual, *V* variance.

### Determination of Trajectories for Perceived Stress

Table 3 presents the model fit statistics for the GMM models concerning stress (i.e., from one to two latent classes). The VLMR-LRT and BLRT were not significant for the two-class model, indicating that the two-class model did not fit the data better than the one-class solution. Although the two-class model had a high entropy, it included a subgroup smaller than 1% of the sample (i.e., it contained only one participant) and the BIC-value was higher than the one-class model. Therefore, the one-class GMM model was chosen as the final model. This class (i.e., whole sample) is characterized by an initial moderate score on stress that remained stable during the pandemic (see Supplementary Fig. 1).

**Table 3:** The model fit information for the GMM models for stress (N = 178).

Classes	AIC	Adjusted BIC	BIC	Class counts (%)	Entropy	VLMR-LRT	BLRT	Parameters
1	432.363	432.483	457.818	–	–	–	–	8
2	434.329	434.493	469.329	1: 1 (0.6); 2: 177 (99.4)	.995	.377	1.000	11

*GMM* Growth Mixture Model, *AIC* Akaike Information Criterion, *BIC* Bayesian Information Criterion, *VLMR-LRT* Vuong-Lo-Mendell-Rubin Likelihood Ratio Test, *BLRT* Bootstrap Likelihood Ratio Test.

### Determination of trajectories for depression and anxiety

**Figure 2:**
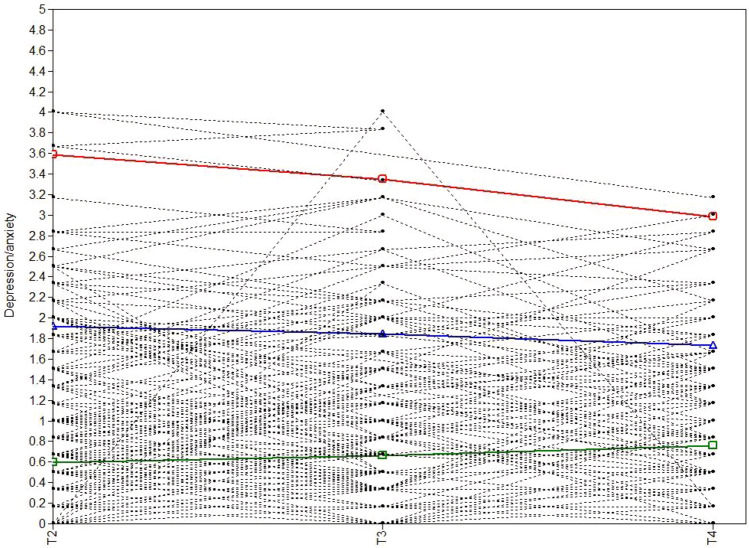
Estimated means and observed individual values during the COVID-19 pandemic for depression and anxiety. Note. The red line is the smallest class (*N* = 5, 3%; high-decreasers), the blue line the intermediate class (*N* = 53, 30%; moderate-stable) and the green line the largest class (*N* = 120, 67%; low-stable).

Table 4 represents the model fit statistics for all GMM models concerning depression and anxiety (i.e., from one to four latent classes). For the GMM models with more than three classes, the variance of the slope was fixed at zero because of the negative definite in the covariance matrices. The BLRT and VLMR-LRT were significant for the three-class model, and the BIC-value was the lowest, indicating that the three-class model fitted the data better than the two-class solution. The three-class model included a small subgroup containing five participants, that is, 3% of the sample. Although 5% is recommended as a minimum group membership probability^32^, subgroups containing more than 1% of the total sample are considered to be acceptable^28^ and are theoretically and clinically interesting. The three-class model was, therefore, chosen as the final model. The largest class, low-stable (*N* = 120, 67%), is characterized by an initial low score on depression and anxiety that remained stable during the pandemic. The intermediate class, moderate-stable (*N* = 53, 30%), is characterized by an initial moderate score on depression and anxiety that remained stable during the pandemic. The smallest class, high-decreasers (*N* = 5, 3%), is characterized by an initial high score on depression and anxiety that significantly decreased during the COVID-19 pandemic. The three observed and estimated trajectories are represented in Fig. 2 and descriptives, intercepts, and slopes of the three classes are demonstrated in Table 6.

**Table 4:** The model fit information for the GMM models for depression and anxiety (N = 178).

Classes	AIC	Adjusted BIC	BIC	Class counts (%)	Entropy	VLMR-LRT	BLRT	Parameters
1	979.409	979.529	1004.864	–	–	–	–	8
2	963.042	963.206	998.041	1: 45 (25.3); 2: 133 (74.7)	.762	.174	< .001	11
3^a^	952.050	952.229	990.231	1: 5 (2.8); 2: 53 (29.8); 3: 120 (67.4)	.854	.035	< .001	12
4^a^	945.881	946.104	993.607	1: 5 (2.8); 2: 24 (13.5); 3: 47 (26.4); 4: 102 (57.3)	.763	.610	.040	15

*GMM* Growth Mixture Model, *AIC* Akaike Information Criterion, *BIC*  Bayesian Information Criterion, *VLMR-LRT* Vuong-Lo-Mendell-Rubin Likelihood Ratio Test, *BLRT* Bootstrap Likelihood Ratio Test.

^a^The variance of the slope was fixed at zero for all classes.

### Predictors of the course of perceived stress

As different trajectories of stress during the COVID-19 pandemic could not be distinguished, we could not perform logistic regression analyses to predict group membership (i.e., the three-step method). Instead, to predict variation in stress, we conducted regression analyses based on the whole-group LGC analyses (see Table 2). We only performed analyses to predict individual intercepts, as the variance of the slope was not significant (Table 2). The prepandemic predictors (i.e., stress, demographics, and psychosocial variables) were simultaneously added to the model. Results demonstrated that several prepandemic factors, that is, stress, gender, living situation, and social support, were related to initial levels of stress (i.e., intercept, see Table 5). Female adolescents and adolescents that do not live with both parents experienced higher initial levels of stress. Lower levels of social support were related to higher initial levels of stress. Furthermore, higher levels of prepandemic stress were related to higher initial levels of stress during the COVID-19 pandemic. Since number of attended sessions (between T1 and T4) positively correlated with stress at T4, we also performed the analyses with addition of this variable as a covariate. Adding number of attended training sessions did not alter the results.

**Table 5:** Results regression analyses of stress for the whole group (N = 178).

	Stress	Age	Gender	Educational level	Ethnic identity	Living situation	Self-esteem	Social support
	B (SE)	B (SE)	B (SE)	B (SE)	B (SE)	B (SE)	B (SE)	B (SE)
Intercept	0.55 (0.07)***	0.04 (0.03)	0.16 (0.05)**	− 0.01 (0.03)	− 0.05 (0.03)	0.13 (0.06)*	− 0.02 (0.06)	− 0.11 (0.05)**

***p < .001; **p < .01; *p < .05.

### Predictors of trajectories of depression and anxiety

Table 6 presents the descriptives of the three latent classes of change in depression and anxiety during the COVID-19 pandemic. Since the high-decreasers trajectory was very small, as it consisted of only five participants, we did not perform predictor analyses for this trajectory. Logistic regression analyses were performed to examine predictors of moderate-stable membership, using moderate-stable membership as the reference (Table 7). Adolescents that did not live with both parents and adolescents who reported lower scores on social support at T1 were more likely in the moderate-stable trajectory. Adding number of attended training sessions did not change the results.

**Table 6:** Descriptives of the three latent classes of depression and anxiety.

	Low-stable (N = 120)	Moderate-stable (*N* = 53)	High-decreasers (*N* = 5)
Intercept (SE)	0.597***	1.918 (0.13)***	3.589 (0.34)***
Linear slope (SE)	0.064 (0.03)	− 0.074 (0.08)	− 0.243 (0.05)***
Depression/anxiety at T1 (*SD*)	0.82 (0.52)	1.63 (0.69)	3.07 (0.67)
Age (*SD*)	13.43 (0.81)	13.60 (0.79)	13.32 (1.40)
Gender: female	49.2%	64.2%	80.0%
**Educational level**			
Pre-vocational	30.0%	34.0%	60.0%
Pre-vocational/senior general	26.7%	22.6%	40.0%
Senior general-pre-university	43.3%	43.4%	0%
**Ethnic identity**			
Western	58.3%	66.0%	60.0%
Mix Western-non-Western	19.2%	17.0%	0%
Non-Western	22.5%	17.0%	40.0%
Living situation: both parents	76.7%	47.2%	60.0%
Self-esteem at T1 (*SD*)	3.02 (0.48)	2.57 (0.50)	1.70 (0.37)
Social support at T1 (*SD*)	3.02 (0.62)	2.53 (0.64)	2.23 (0.75)

***p < .001; **p < .01; *p < .05.

**Table 7:** Results of the multinomial logistic regression analyses for the latent classes of depression and anxiety (N = 177).

	Depression/anxiety		Age		Gender		Educational level		Ethnic identity		Living situation		Self-esteem		Social support	
	OR	B (SE)	OR	B (SE)	OR	B (SE)	OR	B (SE)	OR	B (SE)	OR	B (SE)	OR	B (SE)	OR	B (SE)
Moderate-stable versus Low-stable	0.06	− 2.82 (2.72)	0.65	− 0.43 (0.49)	0.21	− 1.58 (0.99)	0.99	− 0.01 (0.70)	1.65	0.50 (0.40)	0.15*	− 1.88 (0.89)	0.64	− 0.45 (2.51)	5.74*	1.75 (0.80)

*OR* odds ratio.

***p < .001; **p < .01; *p < .05.

## Discussion

The current study aimed to investigate changes in adolescents’ mental health indicators during the COVID-19 pandemic. We explored whether trajectories could be distinguished based on changes in perceived stress and symptoms of depression and anxiety. Results showed that there was no support for distinct trajectories for perceived stress. On average, adolescents experienced stable moderate levels of stress over the course of the pandemic. Since no variation in change over time was identified, we investigated which prepandemic factors (i.e., stress, demographics, and psychosocial variables) predicted individual initial levels of stress. Higher prepandemic levels of stress predicted higher initial levels of stress during the pandemic. Female adolescents and adolescents not living with both parents experienced higher initial levels of stress during the pandemic. Furthermore, adolescents with lower social support before the pandemic experienced higher initial levels of stress during the pandemic. In contrast, results showed three distinct trajectories for depression and anxiety. Most adolescents (67%) reported stable low levels of depression and anxiety during the COVID-19 pandemic, a smaller subgroup (30%) reported stable moderate levels, and a small subgroup (3%) was characterized by initial high levels of depression and anxiety that decreased over the course of the pandemic. Adolescents not living with both parents and adolescents reporting lower social support before the pandemic were more likely to belong to the moderate-stable compared to the low-stable group.

Overall, our results indicate that there was little change in mental health indicators over the course of the pandemic, as the majority of adolescents reported stable levels of perceived stress and symptoms of depression and anxiety. Even in the small subgroup of adolescents with the highest level of depression and anxiety symptoms, who experienced a reduction in depression and anxiety, levels remained fairly high (i.e., higher than the other subgroups) over the course of the pandemic. It could be that these adolescents gained slightly from the lockdowns and restrictions due to less (social) pressure and physical contact (e.g., with friends, teachers) or more free time. Some recent studies during the first COVID-19 lockdown also pointed at improvements in mental health, as lower stress from social isolation was associated with reduced internalizing problems amongst adolescents^33^ and with improved emotional health in female adolescents^34^. Several factors may explain why perceived stress and symptoms of depression and anxiety did not change during the pandemic. First, we followed our participants up until 15 months after the start of the COVID-19 pandemic, which might not be long enough to observe robust changes in mental health indicators, particularly for depression and anxiety symptoms. Changes as a consequence of the pandemic might take more time to appear, especially since the government measures were often subject to change (e.g., in duration or severity). Second, although the situation in the Netherlands differed at the three measurement waves in terms of restrictions and infection rates (see Fig. 1), it could be that adolescents did not perceive any major differences between the three waves, and therefore demonstrated stable courses of perceived stress and symptoms of depression and anxiety during the pandemic. Third, methodological factors might also explain why few differential subgroups with deviating patterns (i.e., increases or decreases) were identified. The low levels of observed variance in perceived stress (between 0.18 and 0.25) and depression and anxiety symptoms (between 0.59 and 0.83) over the course of the pandemic, suggest that potentially existing subgroups with deviating patterns are small. Consequently, a larger sample size would be needed to detect meaningful subgroups based on distinguishable trajectories. Moreover, our data demonstrated various and irregular patterns in individual perceived stress and symptoms of depression and anxiety trajectories (see Supplementary Fig. 1 and Fig. 2), suggesting that adolescents did respond differently over the course of the COVID-19 pandemic. However, these various and irregular patterns, combined with our sample size, may have made it difficult to distinguish deviating patterns experienced by subgroups of adolescents. Finally, it is also possible that the peak of the COVID-19 pandemic, in terms of increases in mental health problems, was primarily at the beginning of the pandemic, after which mental health problems stabilized or reduced. Previous research showing increased mental health problems in adolescents from before to during the pandemic took place in the first months of the pandemic (i.e., between April and July 2020, mostly during the first lockdown)^5,7–9^. Interestingly, recent research at later stages into the pandemic (i.e., from July 2020) suggests that mental health problems of adolescents increased during the first months of the COVID-19 pandemic, but decreased or remained stable later into the pandemic^35–39^, which resembles our results of stable trajectories of perceived stress and symptoms of depression and anxiety during the pandemic (i.e., from September 2020). It could be that adolescents habituated to the (drastic) changes of daily life (in the first months of the pandemic), and therefore showed stable trajectories over the course of this study. In any case, differences between studies in timing of assessments during the COVID-19 pandemic should be acknowledged, as this can affect the interpretation of results.

Our results did not indicate changes in adolescents’ stress levels between 8 and 15 months after the start of the COVID-19 pandemic, as we did not identify trajectories of change nor significant slopes. In contrast, we did observe different trajectories for depression and anxiety. It appears that there is less differentiation between adolescents regarding perceived stress as opposed to symptoms of depression and anxiety during the COVID-19 pandemic, which could be explained by the fact that stress is often faced and experienced by adolescents, and may thus be more common and normative compared to experiencing depression and anxiety. In addition, it might be easier to recognize and talk about feelings of stress relative to depression or anxiety symptoms.

Adolescents facing specific prepandemic vulnerabilities were at increased risk of experiencing more problems during the COVID-19 pandemic. Adolescents with higher prepandemic levels of stress experienced higher initial levels of stress during the pandemic. This is in line with previous research in adolescents that demonstrated that having higher prepandemic stress and depression and anxiety symptoms were associated with higher COVID-19-related concerns and mental distress during the pandemic^6,13^. Female adolescents reported higher initial levels of stress during the pandemic, probably because in general, females are more prone to experiencing internalizing problems^40^. Furthermore, not living with both parents predicted higher initial levels of stress and increased the chance of experiencing moderate (rather than low) levels of depression and anxiety, which is in line with previous research demonstrating that not living with both parents was associated with higher levels of internalizing problems during the COVID-19 pandemic^13,41^. This might be related to a more stressful or unstable home situation, that is more often observed in single-parent households^42^. In addition, higher social support appeared to function as a protective factor for experiencing stress and symptoms of depression and anxiety during the pandemic, as having higher social support increased the chance of reporting lower initial levels of stress and belonging to the low-stable trajectory of depression and anxiety. This is in line with previous research demonstrating that more social support was associated with lower levels of internalizing problems during the COVID-19 pandemic^17^. It could be that contact with peers or family members reduces perceived stress and symptoms of depression and anxiety through shared empathy, talking, or venting. In sum, this study highlighted a number of prepandemic factors associated with experiencing higher initial levels of stress and symptoms of depression and anxiety during the pandemic, including higher prepandemic levels of stress and lower social support. With this knowledge, strategies could be developed to provide individualized and tailored care to support these vulnerable adolescents, for instance enhancing adolescents’ social support.

Previous findings in this sample highlighted that adolescents with specific prepandemic vulnerabilities (i.e., higher stress, maladaptive coping, or internalizing problems) were at increased risk of experiencing more COVID-19-related concerns^6^. Yet, in the present study we mainly observed stable courses of perceived stress and symptoms of depression and anxiety during the pandemic, while more than half of these adolescents reported medium to high COVID-19-related concerns during the pandemic^6^. It appears that some adolescents experience elevated stress levels during the pandemic, but specifically related to the pandemic and not significantly affecting overall stress levels. Specific focus on reducing COVID-19-related stressors could help adolescents cope and deal with the current pandemic.

A limitation of the current study is the low retention rate at the fourth measurement wave (62.2%), which could have influenced or biased the results. Due to the lockdown that was put into effect from December 2020, secondary schools were closed and only partly reopened from March 2021^43^, complicating the data collection. Some students were not reached or did not want to fill in the questionnaire (despite being offered a compensation of six euros after completion), and one school did not want to participate in the fourth measurement wave. Additionally, interpreting the findings in relation to the COVID-19 pandemic and restrictions is complicated, since the measurement waves were not allocated to a specific government measure or lockdown and restrictions changed very frequently (e.g., in duration or severity). Nevertheless, the current study used a longitudinal design with one prepandemic measurement and multiple measurements during the COVID-19 pandemic to identify different trajectories of perceived stress and symptoms of depression and anxiety among adolescents, which provides an unique overview.

Another limitation of the study is the sample size, which was small considering that some observed subgroups contained only few adolescents. Although subgroups containing more than 1% of the total sample are considered to be acceptable^28^, subgroups greater than 5% of the sample are preferred^32^. Yet, small subgroups are theoretically and clinically interesting, as these subgroups often represent adolescents with divergent trajectories compared to the overall sample. In order to examine (smaller) subgroups and increase reliability, future research should examine trajectories of mental health change during the pandemic with larger adolescent samples. In addition, in the current study, we examined depression and anxiety symptoms together instead of separately. It is possible that this could have influenced the results, as previous research in adults observed distinct trajectories for depression and anxiety symptoms^44,45^, and some studies demonstrated increased depressive symptoms, but stable or reduced anxiety symptoms from before to during the COVID-19 pandemic^12,46^. Although our questionnaire demonstrated good reliability on all timepoints, it only contained six items, which might not be enough to fully capture both depression and anxiety symptomology. Future studies should investigate depressive and anxiety symptoms separately.

Finally, adolescents in this study were enrolled in an evaluation study that examined the effectiveness of two short intervention programs (i.e., school-based skills-training programs offered to adolescents who wanted to participate). Number of attended sessions in the skills-training programs was positively associated with reported stress and symptoms of depression and anxiety, indicating that adolescents with higher levels of perceived stress and symptoms of depression and anxiety attended more sessions of the skills-training programs. It is plausible that adolescents who reported more stress and symptoms of depression and anxiety were also more motivated to attend and participate in the programs. It could be that this influenced the results, as half of the participants received (some) help for their perceived problems. Yet, number of attended sessions in the training programs did not alter the results of the predictor analyses. Additionally, it could be that adolescents in this study are not representative for the whole (Dutch) adolescent population, given that our sample only exists of adolescents who registered to participate in a skills-training program. Yet, almost half of the students did not attend any session of the skills-training programs and prepandemic stress levels were comparable to a community sample of adolescents^23^. Moreover, our sample was comparable to Dutch adolescents aged 10–15 years regarding minority background^47^ and educational level^48^, suggesting that our findings are generalizable to community samples of (Dutch) adolescents.

In conclusion, adolescents might not respond to the COVID-19 pandemic to the same extent, or in the same way. In order to better specify how adolescents experience and deal with the pandemic, to ultimately provide more individualized and tailored care for vulnerable adolescents, it is important to examine heterogeneity during the COVID-19 pandemic. The current study, therefore, explored which trajectories were distinguished based on changes in perceived stress and symptoms of depression and anxiety from 8 to 15 months after the start of the COVID-19 pandemic. Results demonstrated that there was no support for distinct trajectories of perceived stress. On average, adolescents reported stable moderate levels of stress during the pandemic. In contrast, three subgroups of adolescents with diverse trajectories of depression and anxiety symptoms were identified. The majority of adolescents reported initial low or moderate levels of depression and anxiety, which remained stable over the course of the pandemic. A small subgroup was characterized by adolescents with high levels of depression and anxiety that decreased during the course of the pandemic. Although this seems promising, levels of depression and anxiety remained fairly high. Overall, our results indicate that there was little change in perceived stress and symptoms of depression and anxiety over the course of the pandemic. Certain prepandemic factors, such as higher stress and lower social support, were associated with higher initial stress or moderate (rather than low) levels of depression and anxiety during the pandemic. Strategies could be developed to provide individualized and tailored care to support adolescents with prepandemic vulnerabilities, for instance programs to enhance adolescents’ social support.

## Supplementary Information

Supplementary Information.

## Data Availability

The datasets generated and analyzed during the present study are available from the corresponding author on reasonable request.
