# Supplementary material for: Trajectories of adolescent perceived stress and symptoms of depression and anxiety during the COVID-19 pandemic
Source: Sci Rep. 2022 Sep 24;12:15957. doi: 10.1038/s41598-022-20344-y (PMC9509354; doi:10.1038/s41598-022-20344-y)

Supplementary Figure 1

Observed individual values and estimated mean (in red) during the COVID-19 pandemic for stress

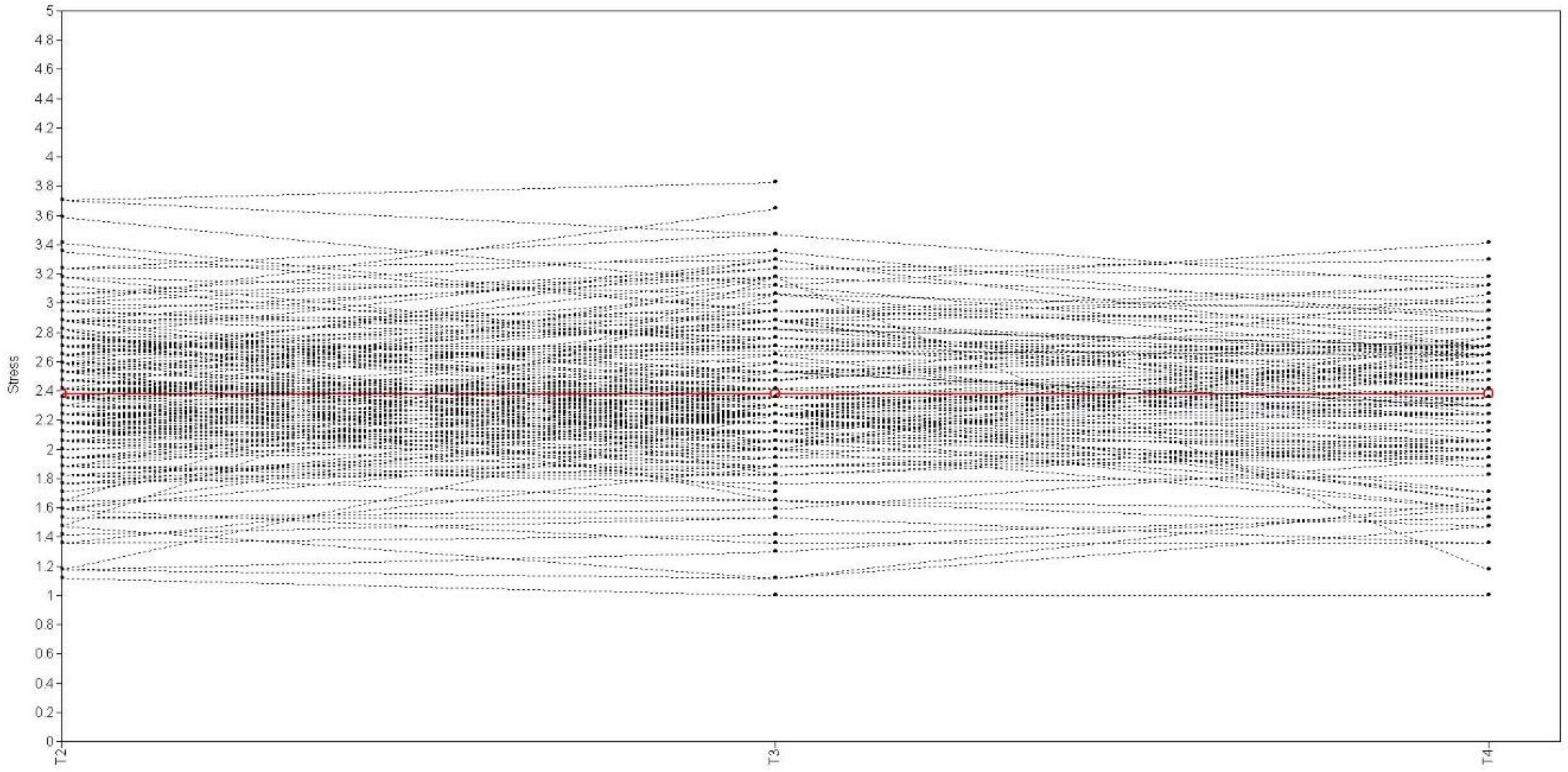

Supplement: Supplementary file 1 — Supplementary Information. [file 41598_2022_20344_MOESM1_ESM.pdf]
